# The concept of folic acid supplementation and its role in prevention of neural tube defect among pregnant women: PRISMA

**DOI:** 10.1097/MD.0000000000038154

**Published:** 2024-05-10

**Authors:** Fikadu Seyoum Tola

**Affiliations:** a Department of Medical Biochemistry, College of Medicine and Health Sciences, Ambo University, Addis Ababa, Ethiopia.

**Keywords:** folic acid, neural tube defect, pregnancy

## Abstract

Folic acid is the synthetic form of vitamin B9, found in supplements and fortified foods, while folate occurs naturally in foods. Folic acid and its derivatives are extremely important in the synthesis of nucleic acids (DNA and ribose nucleic acid [RNA]) and different proteins. It acts as a coenzyme for the transfer of 1 carbon in the biosynthesis of purine, pyrimidine, and amino acids. Folic acid is critically important in rapidly proliferating tissues, including fetus and trophoblastic tissue to prevent neural tube defect (NTD). The main objective of this review is to identify the role of folic acid to prevent NTD among pregnancy mothers. Electronic databases including Web of Science, Google Scholar, MEDLINE, Scopus, and Cochrane library used to systematically search without limitation of publication date and status. In pregnancy, the first trimester is a significant time for neural tube closure. Decreased blood folic acid levels inhibit DNA replication, repair, RNA synthesis, histone and DNA methylation, methionine production, and homocysteine remethylation reactions that cause NTDs in pregnancy. Therefore, folic acid supplementation is critically important for childbearing mothers before conception and in the first trimester pregnancy. As a result, women are recommended to take 400 microgram FA/day from preconception until the end of the first trimester to prevent NTD-affected pregnancies. This allows the developing neural tissue to acquire critical mass and provides the preferred rostrocaudal orientation so that these divisions contribute to the elongation of the developing neural tube in embryos.

## 1. Introduction

### 1.1. Background

Vitamins are the most important organic molecules required by the body in minute amounts for specific functions. The majority of vitamins are not synthesized in the body sufficiently and are therefore obtained from the diet. Folic acid is the synthetic form of vitamin B9, which is found in supplements and fortified foods, while folate occurs naturally in foods. Folic acid is crucial for proper brain functioning and plays an important role in the mental and emotional health of an individual. Additionally, folic acid works closely with vitamin B12 in making red blood cells and helps iron function properly in the body.^[1,2]^

Folic acid and its derivatives are extremely important in the synthesis of nucleic acids (DNA and ribose nucleic acid [RNA]) and different proteins. In this metabolism, folic acid acts as a coenzyme for the transfer of 1 carbon in the biosynthesis of purine, pyrimidine, and amino acids in their respective polymers. It is critically important in rapidly proliferating tissues, including bone marrow tissue, fetal tissue, and trophoblastic tissue. Hence, folate supports rapid growth by enabling DNA synthesis in proliferating cells.^[3]^

DNA methylation is known to control gene expression in different gene classes, like the imprinted gene in the brain cell, the gene of the inactive X chromosome, the germline cell gene, and retrotransposons. It has been shown that one of the cellular processes involved in the normal formation of the neural tube in some vertebrates is oriented cell division. This allows the developing neural tissue to acquire critical mass and provides the preferred rostrocaudal orientation so that these divisions contribute to the elongation of the developing neural tube in embryos. In addition, folate is necessary for amino acid metabolism, neurotransmitter biosynthesis, and phospholipid biosynthesis.^[4]^

## 2. Method

### 2.1. Data source and searching strategies

Electronic databases including Web of Science, Google Scholar, MEDLINE, Scopus, and Cochrane Library were used to systematically search without limitation of publication date and status. Observational, retrospective cohort, prospective case-control, cohort studies, cross-sectional studies, and clinical trials were included.

### 2.2. Inclusion criteria and exclusion criteria

#### 2.2.1. Inclusion

The full articles related to this topic irrespective of study design were included

Studies from all countries were included if published in English to allow for a global understanding of folic acid in pregnant women.

#### 2.2.2. Exclusion

Abstract, lecture, case and review with insufficient information

Studies that reported exclusively non-neural tube defect (NTD) malformations were excluded.

Studies that deal with an association between NTD and other nutrients.

### 2.3. Ethical and dissemination

The protocol of the review does not require ethical approval because it does not involve humans. This article will be published in a peer-reviewed journal and presented at relevant conferences.

### 2.4. Overview of folic acid metabolism

In the human body, folate is absorbed in the mucosal epithelial cells of enterocytes. Folate in foods occurs mainly in the polyglutamate form, which must be hydrolyzed to the monoglutamate form before absorption. This process is carried out by glutamate carboxypeptidase II (GCPII), which exists primarily in the brush border of the proximal part of the jejunum. Mono-glutamate folate is transported across the apical membrane of enterocytes mainly by the proton-coupled folate transporter. Folate in the enterocytes is metabolized to 5-methyl-THF and exported into the portal vein via multidrug resistance-associated protein. It then circulates in the blood and is taken up by the cells via either the reduced folate carrier or receptor-mediated endocytosis of the folate receptors (FRs).^[5]^

Intracellular folate is converted into polyglutamate forms, which are catalyzed by folylpoly-gammaglutamate synthetase. This polyglutamylation is essential for the retention of folate within cells and for the utilization of 1-carbon metabolism. Folic acid biological activity relies on the action of the dihydrofolate reductase (DHFR) enzyme produced in the liver. In order to carry out its biological function, folic acid needs to be reduced by DHFR to dihydrofolate and then to tetrahydrofolate. Then, it is converted to its biologically active form. 5-MTHF.^[1,6]^

**Figure 1.**
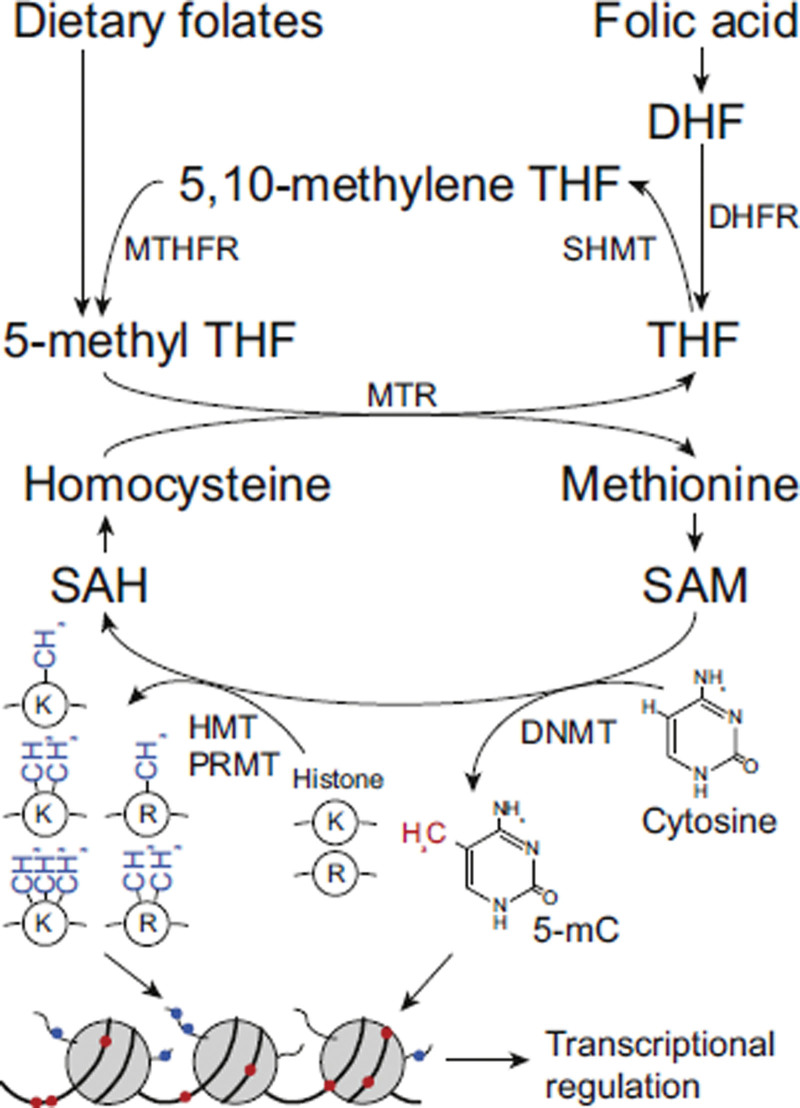
Schematic of folate as a methyl group donor.^[7]^ Either dietary folates or synthetic folic acid are incorporated into the folate 1-carbon metabolism pathway and serve as methyl group donors for various substrates, including protein, DNA, RNA, and lipids. Epigenetic regulation by DNA and histone methylation plays crucial roles in transcriptional regulation and embryonic development. DHFR = dihydrofolate reductase, DNMT = DNA methyl transferase, HMT = histone methyl transferase, K = lysine, mC = methylcytosine, MTHFR = methylenetetrahydrofolate reductase, MTR = methionine synthase, PRMT = protein arginine methyl transferase, R = arginine, SAH = S-adenosylhomocysteine, SAM = S-adenosylmethionine, HMT = serine hydroxymethyltransferase, THF = tetrahydrofolate.^[6]^

The DHFR first converts folate into tetrahydrofolate (THF). Subsequently, with the action of serine hydroxymethyltransferase and 5,10-methylenetetrahydrofolate reductase, THF could be transformed into 5,10-methylenetetrahydrofolate and finally converted to 5-methyl-THF (5-MTHF). The 5-MTHF serves as a transporter to offer an indispensable single-carbon unit for purine and pyrimidine synthesis. Meanwhile, in the methionine synthesis reaction, a methyl group in 5-MTHF is first transferred to VitB12 coenzyme, and then homocysteine is transformed into methionine. The methionine could be further catalyzed by methionine adenosyltransferase (MAT) to produce s-adenosylmethionine, the methyl donor for 5 mC modification (Fig. 1).^[7]^

### 2.5. Rationale of folic acid supplementation in pregnancy

Since folic acid is not synthesized by human tissues, dietary folate should be obtained from food in the form of dihydrofolate, tetrahydrofolate, or 5-methyltetrahydrofolate. These derivatives are naturally present in a variety of foods or as synthetic folic acid added to fortified foods. Folate is specifically important during early embryogenesis, which is characterized by rapid cell divisions.^[8]^ In pregnancy, the first trimester is a significant time for neural tube closure and the formation of brain vesicles; in the second and third trimesters, massive expansion of the brain, accompanied by synaptogenesis and cortex maturation, is mainly occurring. Therefore, continued maternal supplementation of folic acid is still essential for a healthy fetus.^[9]^

There are several factors contributing to the depletion of maternal folic acid. Hormonal change, hemodilution, increased placental and fatal demand, increased maternal catabolism of folic acid as pregnancy proceeds, and periods of rapid cell proliferation that demand high folic acid This low maternal folic acid is associated with several pregnancy-related problems, including inadequate maternal folate status, which has been linked to abruption placentae, preeclampsia, spontaneous abortion, stillbirth, preterm delivery, and low birth weight.^[10]^

Because low folate inhibits DNA replication, repair, RNA synthesis, histone and DNA methylation, methionine production, and homocysteine remethylation reactions. As a result, women are recommended to take 400 microgram FA/day from preconception until the end of the first trimester to prevent NTD-affected pregnancies. All women of childbearing age should take 0.4 mg (400 mg) of folic acid daily when planning a pregnancy.^[11]^

NTDs, including spinal bifida and anencephaly, are the most common birth defects, affecting more than 250,000 pregnancies each year. NTDs are due to improper or incomplete closure of the neural tube during embryogenesis. Mutations in the human PAX3 coding sequence have been identified in some individuals with NTDs. Altered methylation of PAX3 has also been identified in NTD cases, suggesting that altered expression could potentially play a contributing role. Pax3 is a transcription factor that functions to suppress p53-dependent apoptosis in the neuroepithelium.^[12]^

One study found that low blood folate was a significant independent risk after adjustment for a number of known risk factors and other possible confounding variables.^[9]^ Periconceptional use of folic acid supplements reduces the risk of the first occurrence as well as the recurrence of NTDs (relative risk [RR] 0.28, 95% confidence interval [CI] 0.13–0.58).^[5]^

Some studies have suggested that supplementation with folic acid may also reduce the risk of structural cardiac and craniofacial abnormalities. In women at high risk of fetal NTDs (due to previous pregnancy with NTD), a randomized double-blind prevention trial has shown that a higher dose of folic acid supplementation (4 mg/day) reduces the risk of a subsequent NTD-affected pregnancy by 72%.^[1,5]^

According to a study from McGill University Health Centre, methylenetetrahydrofolate reductase deficiency is associated with reduced circulatory folate and reduced methylation potential. When folate metabolism is disturbed, choline can be used as an alternate methyl donor through the generation of betaine, which donates its methyl group for methionine synthesis, thus facilitating S-adenosylmethionine synthesis in a folate-independent manner. When folate metabolism is compromised, the alternate pathway is upregulated, affecting choline pools. Choline is the precursor for many important phospholipids and for the neurotransmitter acetylcholine. Altered levels of these choline-derived metabolites are associated with cognitive impairment, attention and deficits.^[7]^

Therefore, the WHO recommends that all pregnant women in areas with a high prevalence of malnutrition receive 30 to 60 mg of elemental iron, together with 400 micrograms of folic acid, beginning as early as possible during pregnancy and continuing until the birth of the child. Iron supplementation should be accompanied by advice regarding appropriate dietary measures. Consistent with the WHO recommendations, maternal healthcare guidelines recommend that all pregnant women receive daily supplements of 60 mg of elementary iron and 400 micrograms of folic acid, together with vitamin C. An additional recommendation is that all pregnant women receive dietary advice regarding iron-rich foods, including animal and non-animal sources of dietary iron; the importance of consuming dietary iron together with citrus fruit or juice; and the avoidance of concomitant tea or milk consumption with iron, as this inhibits iron absorption.^[13]^

## 3. Conclusion

In pregnancy, the first trimester is a significant time for neural tube closure and the formation of brain vesicles; in the second and third trimesters, massive expansion of the brain is accompanied by synaptogenesis and cortex maturation. Decreased blood folic acid levels inhibit DNA replication, repair, RNA synthesis, histone and DNA methylation, methionine production, and homocysteine remethylation reactions that cause NTDs in pregnancy. There are several factors contributing to the depletion of maternal folic acid. Hormonal change, hemodilution, increased placental and fatal demand, increased maternal catabolism of folic acid as pregnancy proceeds, and periods of rapid cell proliferation that demand high folic acid.

This low maternal folic acid is associated with several pregnancy-related problems, including NTDs, abruption placentae, preeclampsia, spontaneous abortion, stillbirth, preterm delivery, and low birth weight. NTDs, including spinal bifida and anencephaly, are the most common birth defects, affecting more than 250,000 pregnancies each year. NTDs are due to improper or incomplete closure of the neural tube during embryogenesis. As a result, women are recommended to take 400 microgram FA/day from preconception until the end of the first trimester to prevent NTD-affected pregnancies. This allows the developing neural tissue to acquire critical mass and provides the preferred rostrocaudal orientation so that these divisions contribute to the elongation of the developing neural tube in embryos.

## Limitation of the study

The limitation of this review is it considers studies published from both developing and developed countries. The study design is not selectively considered.

## Author contributions

**Conceptualization:** Fikadu Seyoum Tola.

**Data curation:** Fikadu Seyoum Tola.

**Resources:** Fikadu Seyoum Tola.

**Software:** Fikadu Seyoum Tola.

**Validation:** Fikadu Seyoum Tola.

**Visualization:** Fikadu Seyoum Tola.

**Writing – original draft:** Fikadu Seyoum Tola.

**Writing – review & editing:** Fikadu Seyoum Tola.
